# Why does the Aβ peptide of Alzheimer share structural similarity with antimicrobial peptides?

**DOI:** 10.1038/s42003-020-0865-9

**Published:** 2020-03-19

**Authors:** Annalisa Pastore, Francesco Raimondi, Lawrence Rajendran, Piero Andrea Temussi

**Affiliations:** 1 UK-Dementia Research Institute (UK-DRI) at King’s College London, London, UK; 2 The Maurice Wohl Institute of King’s College London, 5 Cutcombe Road, SE5 9RT London, UK; 3 Scuola Normale Superiore, Piazza dei Cavalieri, Pisa, Italy; 4 Dipartimento di Scienze Chimiche, Universita’ di Napoli Federico II, Napoli, Italy

**Keywords:** Biochemistry, Neurochemistry, Structural biology, Peptides, Immunochemistry

## Abstract

The Aβ peptides causally associated with Alzheimer disease have been seen as seemingly purposeless species produced by intramembrane cleavage under both physiological and pathological conditions. However, it has been increasingly suggested that they could instead constitute an ancient, highly conserved effector component of our innate immune system, dedicated to protecting the brain against microbial attacks. In this antimicrobial protection hypothesis, Aβ aggregation would switch from an abnormal stochastic event to a dysregulated innate immune response. In this perspective, we approach the problem from a different and complementary perspective by comparing the structure and sequence of Aβ(1-42) with those of bona fide antimicrobial peptides. We demonstrate that Aβ(1-42) bears convincing structural similarities with both viral fusion domains and antimicrobial peptides, as well as sequence similarities with a specific family of bacterial bacteriocins. We suggest a model of the mechanism by which Aβ peptides could elicit the immune response against microbes.

## Abstract (web summary)

Pastore et al. provide independent evidence that the Alzheimer Aβ peptides could function as antimicrobial peptides based on convincing structural and sequence similarities with viral fusion domains and established antimicrobial peptides. Aβ could dispatch an antimicrobial function through a mechanism that involves membrane pore formation.

## Introduction

Aβ peptides are at the root of the pathology of Alzheimer disease (AD)^1^, one of the devastating diseases of our increasingly ageing society. The peptides originate from the action of specific proteases, called secretases, on the amyloid precursor protein (APP) inside the membrane of neuronal cells. Sequential cleavage by β-(BACE) and γ-secretase produces different peptides ranging from 1–37 to 1–43 amyloid-β peptide fragments, with 1–40 being the most abundant and 1–42 being the most aggregation prone and more toxic. AD mostly affects people over 65 years of age, but the non-symptomatic phase might last several decades, during which the peptides form large aggregates (plaques) which contain amyloid fibrils of Aβ peptides^2^.

The aggregates first observed by Alois Alzheimer in 1906 were originally regarded as the culprits of AD leading to what is now commonly called the amyloid hypothesis^1^. The traditional formulation of the amyloid hypothesis points to the cytotoxicity of mature aggregated amyloid fibrils, which are believed to be the toxic form of the protein responsible for disrupting the cellular calcium ion homoeostasis, thus inducing apoptosis. After the original upraising of the amyloid hypothesis, however, it started to become progressively clear that toxicity of the final aggregates was at most marginal: fibrils could be absent or sparse in advanced AD patients or dense in mild patients or healthy individuals. The culprit changed, and the main cause of cell damage was attributed to Aβ oligomers or, more likely, to an ensemble of oligomers of different stoichiometries and/or morphologies formed during the process of aggregation^3^. A drastically different alternative views Aβ monomers as potential agents capable of damaging membranes. Indeed, there is a vast literature that has studied the effects of Aβ peptides on the membranes and how they affect their properties^4^.

Almost two decades ago, we suggested that Aβ might function as a viral peptide^5^ based on sequence and structure similarities with the influenza virus fusion domain^6^, hinting at a pore-formation (hereafter referred to as poration) mechanism of interaction^7^. Shortly after, the structure of the influenza virus fusion domain allowed suggestion of a detailed model for the membrane poration mechanism^8^. It could be easily foreseen that the ability to perforate membranes could be used against own brain cells but also against invasive cells, a feature reminiscent not only of viruses but, more in general, of antimicrobial peptides (AMPs).

The “viral-like nature hypothesis” was not followed up by others, but during the last 9 years it was repeatedly suggested that Aβ peptides may act as AMPs^9^. Both this suggestion and the viral hypothesis open a completely different perspective to the role of Aβ and directly links neurodegeneration to immunology. Under this hypothesis, Aβ would not be a peptide released into the cell by accident or genetic predisposition, but a specific and generalised response to foreign agents^10^. This “Antimicrobial Protection Hypothesis” would thus significantly change the paradigm, and identify Aβ and its aggregation as the extreme conclusion of a long chain of events most of which would be a natural response of our immunological system to intruders. Under this hypothesis, Aβ would, in other words, return to the stage playing an essential element of the neuronal well-being rather than being a foe.

Here, we revisit the membrane poration hypothesis of Aβ and propose new important aspects that could support the AMP hypothesis: we focus on a completely underemphasised perspective that compares the sequence and structural aspects of Aβ peptides with those of other AMPs. We do hope that this work will be inspirational to other researchers and suggest new avenues to approach the role of Aβ in AD.

### An immunologic point of view of AD

The idea that infection could play a relevant role in the pathogenesis of AD was already proposed in the early years of AD research, but abandoned soon after^11^. The first paper showing experimental evidence in favour of an antimicrobial activity of Aβ was published in 2010^12^ demonstrating that Aβ peptides have an efficacy comparable to that of the well-known human AMP LL-37. Early studies on Aβ as an AMP were summarised in an insightful review^13^, where the authors stated that the data published after 2010^13–15^ showed convincingly that Aβ peptides can indeed act as AMPs, killing clinically relevant microbes. The paradigm of Aβ as an AMP was taken a step forward by Fulop et al.^16^, who proposed explicitly that Aβ may act as a natural defence against infections which becomes a menace only when the inflammation becomes chronic. The fact that Aβ behaves as an AMP adds credence to an infection origin in the aetiology of AD. It was independently noticed that transgenic mice raised in conventional husbandry develop neurodegeneration more quickly than if raised in pathogen free conditions^17^. While this observation could simply be explained as the consequence of better conditions, it could be suggestive, together with other evidence of a correlation between infection and disease. Different groups were able to relate the defence mechanism to aspects of the link between infection and senescence and demonstrated an increase in bacterial populations in Alzheimer brain tissue compared with normal^18^. The discovery of pathogens in AD patients’ brains hints at the emergence of a link between microbiota and senescence^19^. Gingipain, a *Porphyromonas gingivalis* toxin, was detected not only in the brains of people deceased by AD but also in the brains of old people who died prior to developing AD^20^.

### AMPs and their structure

AMPs, as crucial components of the innate immune system, are able to kill a variety of microbes, including bacteria, viruses, fungi and yeasts. The first reported AMPs were probably those described by Zeya and Spitznagel as a family of low-molecular-weight cationic peptides with selective antimicrobial activity^20–22^.

Most AMPs have short sequences rich of positively charged residues and consist only of amphipathic or hydrophobic helices^23^. Others are cyclic peptides or mini-proteins with β-sheet structures. Since the purpose of this review is the comparison of Aβ with bona fide AMPs which share structural similarities, we shall restrict our structural comparison to linear peptides that have highly helical conformations in apolar media^5,24–26^ with structures determined at a high resolution. Short linear peptides are generally disordered in aqueous solutions, but adopt helical conformations in environments that mimic the interior of membranes^23,27^.

### Influenza HA virus fusion domain

**Fig. 1:**
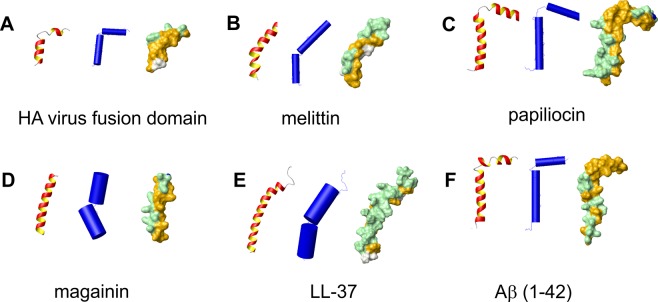
Comparison of the structures of Aβ(1–42) with those of selected helical antimicrobial peptides. All structures are represented left to right as ribbon, schematic secondary structure in which helices are depicted as cylinders and a surface picture enhancing the distribution of polar (green) and apolar (orange) residues. **a** HA virus fusion domain (1ibn); **b** melittin (2mlt); **c** papiliocin (2la2); **d** magainin (2mag);(**e** LL-37 (2k6o) and **f** Aβ(1–42) determined by NMR in an aqueous mixture of hexafluoroisopropanol (pdb id 1iyt)^5^. Structures were generated using MOLMOL^52^.

Enveloped viruses infect cells by fusion between the membranes of the virus and of the cell. Fusion is promoted by envelope proteins whose common feature is a highly conserved hydrophobic fusion domain, generally located at the N-terminus of the protein, and very sensitive to even conservative point mutations^28^. The structure of the fusion domain of influenza HA virus was solved by NMR in deuterated DPC micelles at pH 5, which favours fusion, (pdb 1ibn) and at pH 7.4 (pdb 1ibo). At both pH values, the domain is characterised by two amphipathic helices at ~90° connected by a kink (Fig. 1a). Its sequence does not contain basic residues.

### Melittin

This peptide is not often classified as an AMP because it comes from bee venom but it also inserts into membranes and produces poration. It was recently included among the AMPs from a structural comparison^9^. Melittin is amphiphilic with five positively charged residues and ten hydrophobic residues. The structure of melittin (Fig. 1b) was solved in the solid state (pdb 2mlt)^29^ and in a methanol solution^30^. The peptide is tetrameric in the crystal in agreement with the data in aqueous solutions at high ionic strength^31^. The monomer in both the crystal and the solution structures consists of two helices interrupted around a proline, with a different kink angle between the helices. This is close to 50° in the crystal, and 20° in solution.

### Papiliocin

Cecropins, originally found in the haemolymph of *Hyalophora cecropia*, are peptides of 31–37 residues and active against bacteria. They constitute the essential part of the innate immune system of insects. We analysed the structure of another member of the family, papiliocin that is a cecropin-like peptide found in the Asian butterfly Papilio xuthus. Papiliocin has nine basic and eighteen strongly apolar residues out of 37. The structure of papiliocin in DPC micelles (pdb 2la2)^32^ is composed of two helices that form an angle >90° (Fig. 1c).

### Magainin

Magainins are one of the first families of AMPs discovered in *Xenopus laevis*^33,34^. They act on the membranes of many microbes, probably by forming pores. Magainin 2, the best studied in the magainin family, is a peptide of 23 residues that contains five basic and ten strongly apolar residues. The structure of magainin (pdb 2mag)^35^ determined in perdeuterated dodecylphosphocholine (DPC) and sodium dodecylsulfate micelles is composed of two amphipathic helices at a small angle (Fig. 1d).

### LL-37

The two main groups of AMPs found in mammals are defensins and cathelicidin derivatives. We will not consider defensins because they have a rigid architecture stabilised by several disulphide bonds. The cathelicidin family belongs to the cystatin superfamily of proteins. Cathelicidins act as precursor molecules that, after proteolysis, release a linear AMP. The most important human AMP derived from cathecilidin is LL-37, a highly basic amphipathic peptide of 37 residues with 11 basic and 13 strongly apolar residues. The solution structure^36^ consists of two helices spanning residues 2–31, with a kink between residues 14 and 16 (Fig. 1e).

### Structural comparison with Aβ(1–42)

If we now compare these peptides with Aβ(1–42), we find interesting similarities. Among the several structures available, we selected that (pdb 1iyt)^5^ in a mixed (20/80) water/hexafluoroisopropanol solution, which is a medium that mimics the membrane environment^37^. The structure consists of two helices at an angle close to 90° and encompassing residues 8–25 and 28–38, with the connecting link adopting a regular type I β-turn (Fig. 1f). Among the AMPs analysed here, the structure most similar to Aβ is that of papiliocin: the tilt angle between the helices is similar and the surfaces, particularly in the C-terminus, have a similar distribution of apolar residues. The structure of the influenza virus fusion domain is also similar to the C-terminus of Aβ(1–42)^5^. In particular, the angle between the helices is almost identical and the distribution of residues makes the concave surface of both structures apolar.

### Exploring the sequence space

**Fig. 2:**
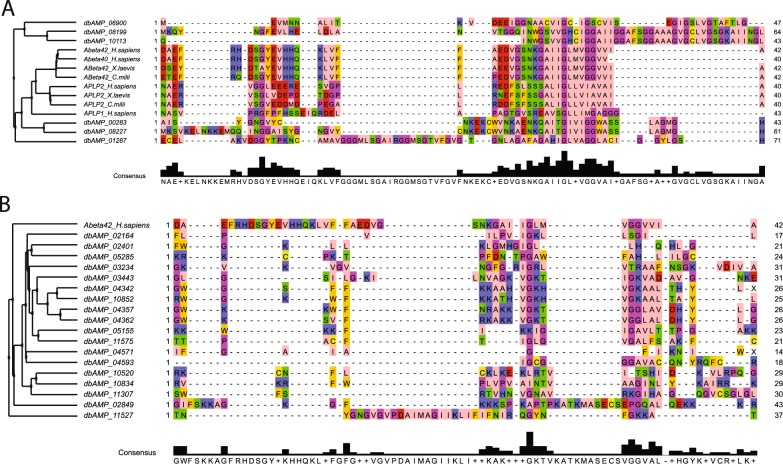
Sequence analysis of Aβ. **a** Multiple sequence alignment of PSIBLAST hits from dbAMP and *APP* and *APLP1/2* homologue sequences from *H. sapiens*, *X. laevis* and *C. milii*. **a** Multiple sequence alignment, generated through Expresso/Tcoffee (http://tcoffee.crg.cat/)^53^, using available structural information fetched from the PDB to refine the alignment. Visualisation has been done through Jalview^54^, using the Zappo colour scheme to highlight amino acid physico-chemical properties and the average distance method to calculate the dendrogram. We excluded from the alignment the sequences dbAMP_07565 and dbAMP_08871, as their lengths prevented creating a compact representation. **b** Sequence similarity search through dbAMP. Multiple sequence alignment of 19 representative, validated AMPs sequences filtered accordingly to sequence features and Aβ(1–42) peptide. Alignment was generated and rendered as in panel **a**, and sequences dbAMP_01836 and dbAMP_12362 were excluded to achieve a more compact representation.

We then explored dbAMP (http://140.138.77.240/~dbamp/index.php, v1.4), a comprehensive meta-database that collects peptides with reported antimicrobial activity, to search for sequence similarities with Aβ(1–42). Searches were performed by PSIBLAST^38^, using ten iterations, turning off composition-based statistics and filtering out low complexity regions. This revealed ten sequences with significant matches (*e*-value < 10) to Aβ(1–42) (Fig. 2; Supplementary Table 1a), half of which are experimentally validated AMPs. Notably, also Aβ(1–42) and Aβ(1–40) are reported in dbAMP as validated AMPs. Three validated AMPs gave significant matches to Aβ(1–42): the Bacteriocin carnobacteriocin BM1 (dbAMP_00283, gene: *cbnBM1*, Uniprot ID: CBB1_CARML) and two other bacteriocines not yet reviewed in Swissprot (dbAMP_01287, gene: *blp1a*, Uniprot ID: I0B595_9LACO and dbAMP_06900, gene: BACERE00183_06588, Uniprot ID: A0A1N7URV8_BACCE) (Supplementary Table 1a). Multiple alignment of these sequences revealed conserved patterns flanking the region that hosts the kink in Aβ(1–42). We identified an invariant sequence signature (i.e., G*XXX*GG, where *X* is an apolar amino acid in most of the sequences including Aβ(1–42)) at the peptide C-terminus, and a conserved motif (i.e., *XXXX*N*X*G) N-terminally preceding the kink. Interestingly, the corresponding peptides of close *APP* homologues such as *APLP1/2*, which are characterised by an incomplete *GXXXGG* motif (Fig. 2a), are not reported by all prediction programmes as having antimicrobial activity.

As an additional and independent test, we inspected the sequence similarity of Aβ(1–42) with peptides from dbAMP which were experimentally validated irrespective of PSIBLAST similarity searches. We restricted the analysis to sequences having generic features similar to those of Aβ(1–42), i.e., being shorter than 50 amino acids in length, having less than four cysteines (to avoid considering AMPs containing β-hairpins) and matching to Swissprot protein regions annotated as having a transmembrane topology or being localised within the membrane (Supplementary Table 1b). This led to a total of 27 sequences, which were clustered following the CD-HIT approach^39^ to yield a pool of 21 representatives (from 15 source organisms, out of which 7 bacterial). Despite higher sequence divergence, multiple sequence alignment revealed that the G*XXX*GG motif is the most conserved feature also in this pool, suggesting that this is a universal signature of this particular type of AMPs (Fig. 2b; Supplementary Table 1c, d).

We can thus conclude that, based on sequence, Aβ peptides seem to share homology with a specific family of bacteriocins.

### Mechanism of action

How could Aβ be beneficial against infected cells by its membrane penetrating ability? The majority of AMPs interact with the membrane directly without targeting specific receptors. Thanks to electrostatic and hydrophobic interactions, the peptide concentration on the bacterial membrane increases to reach a critical value that eventually promotes membrane penetration according to pore or non-pore forming models. Some peptides that exhibit a random coil structure at neutral pH undergo a conformational transition at lower pH that results in an enhanced interaction with the membrane. This interaction promotes the formation of pores with consequent lysis of the membrane bilayers. The N-terminal peptide region of the hemagglutinin subunit of influenza, for instance, contains such a fusogenic sequence.

**Fig. 3:**
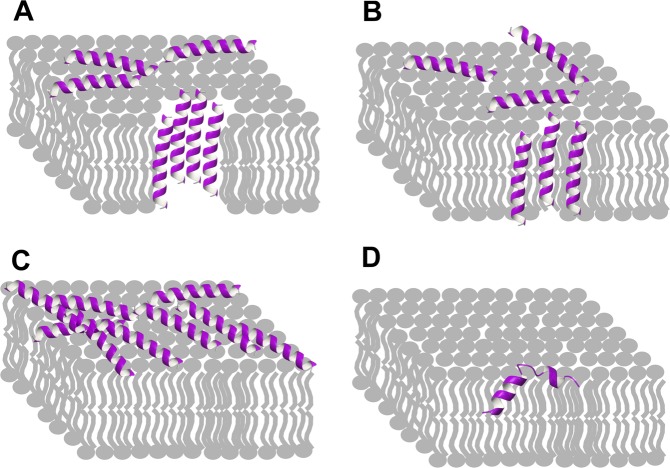
Main mechanisms of action proposed for AMPs. **a** Barrel-stave model: helices of an AMP (violet) associate to form a pore; **b** toroidal model: helices interleave with lipids along the wall of the pore; **c** carpet model: helices cover the membrane surface as a carpet; **d** boomerang model: the kink joining two helices is instrumental for membrane penetration, only one of several boomerangs is shown.

Pore models are often subdivided into the barrel-stave and toroidal models (Fig. 3a, b). In all of them the peptides are linear, without disulphide bridges and form one or more helices. The rare barrel-stave model, mainly represented by alamethicin, requires that amphipathic peptides assemble first on the surface of the membrane and successively insert perpendicularly through the lipid bilayer forming lateral peptide–peptide interactions. Toroidal models do not require specific peptide–peptide interactions, because the channel wall is formed both by peptide helices and lipids^40^. Examples are melittin and magainin. Another popular mode of interaction with membranes, which does not require pore formation, is the so-called “carpet model”^41^, in which the peptides are adsorbed by the lipid bilayer until they cover the entire membrane, i.e., forming a “carpet” (Fig. 3c). Unfavourable interactions with the membrane surface eventually affect membrane integrity, leading to disruption accompanied by micelle formation.

Finally, a “boomerang model” was proposed for viral fusion domains (Fig. 3d) which undergo a conformational change at acidic pH leading to a V-shaped (boomerang) structure characterised by a kink^6^. Based on the structure of Aβ, we propose that this boomerang model could be crucial for its behaviour as an AMP.

Several viral peptides have been shown to directly ferry cargoes across the plasma membrane^42^. Examples include HIV-tat, the transcription factor Antennapedia, the VP22 protein from herpes simplex virus^43^. Their peptidic regions responsible for cell penetration are either amphipathic (model amphipathic peptides or transportan) or arginine (Tat, VP22, penetratin) containing stretches of ~30 amino acids^42^. While the exact mechanism through which these peptides ferry either themselves or their conjugated payloads is unclear, it is indeed possible that their electrostatic interaction with the phospholipids might trigger the conformational change that allows insertion of these peptides into the membrane^44^. Lipids of the host’s membrane or subcellular organellar membrane or the microbial membrane could be of paramount importance in the process. Several studies show that while some peptides could traverse the membrane through lipid interactions, conjugates of such peptides are mainly internalised via endocytosis^45–47^. Akin to the influenza HA peptide or adenovirus capsid protein, Aβ could create havoc to the bacterial membrane and endosomal membranes of the infected cells. Several lipids, including the raft lipids GM1 and POPG, mediate the interaction both with the head groups and the tail^48^. Once internalised, Aβ could, in its native form or a particular strain (GM1-Aβ, oligomeric Aβ), poke holes in the endosomal lipid bilayer rupturing the endosomal membrane^49,50^ through endo-osmolysis—wherein the cytoplasmic contents flow into the endosome causing rupture of the endosomal membrane. This would cause apoptosis or necroptosis of the infected cell, thus conferring protection against the infected cell and the microbe.

## Conclusions

We have demonstrated  here that both the sequence and the structure of the Aβ(1–42) peptide have strong similarities with AMPs from various organisms. Aβ peptides have been considered for a long time functionless byproducts of APP catabolism. This view mostly emerged because, when Aβ was first identified, intramembrane cleavage was seen as an abnormal catabolic pathway. As a consequence, the production of Aβ was exclusively associated to a pathologic state. It is now accepted that intramembrane cleavage is a normal proteolytic pathway that generates different functionally important peptides. Aβ could thus be a normal constitutively generated peptide found in neurons. In support to this hypothesis is the evidence that the sequence of this region of APP is 100% conserved throughout most vertebrates^51^.

The assumption of Aβ(1–42) as an AMP provides a logical explanation for the presence of this peptide in our body and its concentration in neuronal synapses. It finally attributes an important and well-defined role to the peptide. In this scenario, Aβ peptides would be important components of the nervous system with several different functions, one of which being that as AMPs specialised in protecting the brain from foreigner attacks. Their release by the secretases would thus not be a mere accident that occurs only in concomitance with mutations. The experimental evidence in support of this thesis is getting increasingly convincing (there are for instance 28 reviews in Pubmed on “Aβ and antimicrobial peptide”, 16 of which published within the last 5 years).

Our findings are in turn compatible with several interesting not mutually exclusive scenarios. Based on the structural and sequence similarity with validated AMPs, we could for instance envisage that prolonged infection of bacteria (for instance from food but not only) that release Aβ-like bacteriocins could bring our immune system to develop anti-Aβ antibodies. Meanwhile neurons would develop Aβ as a physiologic important AMP to combat the bacterial attack. The similarity between Aβ and Aβ-like peptides would bring the immune response to turn its action against the neurons determining an autoimmune response and tissue damage. Alternatively, microbial Aβ-like peptides could favour Aβ oligomerization and provide a template for aberrant aggregation according to a prion-like mechanism. These and other hypotheses will need careful experimental validation which should lead to a better understanding of the AD aetiology.

In conclusion, the antimicrobial hypothesis^10^ proposes a completely different approach to AD from that so far adopted while not changing the nature of the molecular culprit: instead of eliminating Aβ or prevent its cleavage, an alternative strategy would be to identify possible preferential sources of infection. If this were possible, we could envisage to design vaccines to immunise people against these agents. Potential targets could for instance be proteins from *Porphyromonas gingivalis*, which has been suggested to be related to AD^20^. A better understanding of the human microbiome might be essential to inform these studies.

## Methods

Structures were retrieved from PDB (https://www.rcsb.org/) and visualised by the MolMol software (https://sourceforge.net/projects/molmol/). Sequence similarity searches were carried out using PSIBLAST (https://www.ebi.ac.uk/Tools/sss/psiblast/). Multiple sequence alignments were generated by Expresso/TCoffee (http://tcoffee.crg.cat/apps/tcoffee/do:expresso) and visualised through Jalview (http://www.jalview.org/getdown/release/).

## Supplementary information

Supplementary Information

## Data Availability

The data sets analysed in the current study are available in the dbAMP repository (10.1093/nar/gky1030). These data sets were derived from the following public domain resources: http://140.138.77.240/~dbamp/index.php.

## Code availability

All software used in this paper is freely available on the web.
