# Supplementary material for: Why does the Aβ peptide of Alzheimer share structural similarity with antimicrobial peptides?
Source: Commun Biol. 2020 Mar 19;3:135. doi: 10.1038/s42003-020-0865-9 (PMC7081199; doi:10.1038/s42003-020-0865-9)
Supplement: Supplementary file 1 — Supplementary Information [file 42003_2020_865_MOESM1_ESM.pdf]

Supplementary Materials
The following tables have been simplied and shortened. To get the complete PSIBLAST output please do refer to the main text Availability Statement.

Supplementary Table 1A: AMPs from dbAMP producing significant hits in PSIBLAST searches of Abeta 42 peptide

dbAMP_ID	SEQUENCE	Length	Activity	dbAMPvalid	Top_Sprot_Blast_ Match(Uniprot ID)	E-value	Identity
dbAMP_07565	MIVKIAILVIAITFNDVSAKTSYKICVPSQFMKACEQMLEVETKSKAILECLPARDRVECLTLVQQRQADLVPVDPEDMYVASKLPNQDFVLFQEFRTDEEPDAEFRYEAVIVVHKDLPVTNLDQLKGLKSCHTGINRNVGYKIPLTMLMKRSVFPAMTDRSISPKENELKALSTFFSKSCIVGQWSPDPKTNTFWKSQSSKLCSMCEDPAKCDYPDNYSGYEGALRCLAHNGGDVAFTKVIYVRKFFGLPVGTS	255	Antimicrobial	nonValidated	TRF_MANSE	2.00E-150	79
dbAMP_08227	MKSVKELNKKEMQQINGGAISYGNGVYCNKEKCWVNKAENKQAITGIVIGGWASSLAGMGH	61	Antimicrobial	nonValidated	CBB1_CARML	1.00E-37	100
dbAMP_08871	MNTKTMSQFEIMDTEMLACVEGGGCNWGDFAKAGVGGGAARGLQLGIKTRTWQGAATGAVGGAILGGVAYAATCWW	76		nonValidated	ZN619_HUMAN	3.1	32
dbAMP_00283	AISYGNGVYCNKEKCWVNKAENKQAITGIVIGGWASSLAGMGH	43	Antibacterial, AntiGram_p, AntiGram_n, Antimicrobial	Validated	CBB1_CARML	8.00E-25	100
dbAMP_08199	MKQYNGFEVLHELDLANVTGGQINWGSVVGHCIGGAIIGGAFSGGAAAGVGCLVGSGKAIINGL	64		nonValidated	LAFA_LACJO	0.004	46
dbAMP_00972	DAEFRHDSGYEVHHQKLVFFAEDVGSNKGAIIGLMVGGVV	40	Antibacterial, AntiGram_p, AntiGram_n, Antifungal, Antiviral	Validated	A4_HUMAN	8.00E-23	100
dbAMP_00973	DAEFRHDSGYEVHHQKLVFFAEDVGSNKGAIIGLMVGGVVIA	42	Antibacterial, AntiGram_p, AntiGram_n, Antifungal, Antiviral	Validated	A4_HUMAN	4.00E-24	100
dbAMP_10113	QINWGSVVGHCIGGAIIGGAFSGGAAAGVGCLVGSGKAIINGL	43		nonValidated	PLST2_ARATH	0.22	58
dbAMP_01	ECELAKVDGGYTPKNCAMAVGGGMLSGAIRGGMS	71	AntiGram_p Antimicrobial	Validated	SURE_ACTSZ	3	37

287	GTVFGVGTGNLAGAFAGAHIGLVAGGLACIGGYLGSH						
dbAMP_06900	MEVMNNALITKVDEEIGGNAACVIGCIGSCVISEGIGSLVGTAFTLG	47		Validated	ADH7_RAT	6.6	37

Supplementary Table 1B: Representative (first 100 top hits), validated AMPs from dbAMP filtered according general sequence features

dbAMP_ID	SEQUENCE	Length	Activity	dbAMPUniprot	dbAMP_Taxonomy
dbAMP_01836	FKKKKRNIGTFVFFAIALFCTVMFAYLLLTNQY VPIDYNVPRYA	44	AntiGram_p,=		Bacteria,Unclassified,Firmicutes,Bacilli, Lactobacillales,Streptococcaceae,Lactococcus, Lactococcus lactis
dbAMP_02164	FLPVILPVIGKLLSGIL	17	Antibacterial, AntiGram_p, MammalianCells		Eukaryota,Metazoa,Chordata, Amphibia, Anura, Ranidae,Rana,Rana sakuraii&&Animalia, Amphibians
dbAMP_02401	FWGKLLKLGMHGIGLLHQHLG	21	Antibacterial, AntiGram_p, AntiGram_n, Antifungal, AntiMRSA	Q7SZG9	Eukaryota,Metazoa,Chordata,Craniata,Vertebrata, Euteleostomi, Actinopterygii,Neopterygii,Teleostei, Neoteleostei, Acanthomorphata,Carangaria, Pleuronectiformes,Pleuronectoidei,Pleuronectida, Glyptocephalus.&Animalia, Pisces
dbAMP_02849	GIFSKKAGKGFKKKSPKAPTPKATKMASECSE PGQALQEKKKR	43			Eukaryota,Metazoa,Chordata, Aves,Columbiformes, Columbidae,Columba,Columba livia
dbAMP_03234	GKVKVGVNGFGRIGRLVTRAAFNSGKVDIVA	31	Antibacterial, Antifungal		Eukaryota,Viridiplantae,Streptophyta,Unclassified, Brassicales,Brassicaceae, Arabidopsis, Arabidopsis thaliana&&Animalia, Mammals
dbAMP_03443	GLGSILGKILNVAGKVGKTIGKVADAVGNKE	31	Antibacterial, AntiGram_n		Eukaryota,Viridiplantae,Streptophyta,Unclassified, Brassicales,Brassicaceae,Heliophila,Heliophila coronopifolia&&Animalia, Amphibians
dbAMP_04342	GWGSFFKKAAHVGKHVGKAALTHYLX	26	Antibacterial,	P81941	Eukaryota,Metazoa,Chordata,Craniata,Vertebrata, Euteleostomi, Actinopterygii,Neopterygii,Teleostei ,Neoteleostei, Acanthomorphata,Carangaria, Pleuronectiformes,Pleuronectoidei,Pleuronectida, Pseudopleuronectes.
dbAMP_04357	GWKKWFNRAKKVGKTVGGLAVDHYLG	26	Antibacterial, AntiGram_p, AntiGram_n, Antifungal, AntiMRSA	Q7SZH3	Eukaryota,Metazoa,Chordata,Craniata,Vertebrata, Euteleostomi, Actinopterygii,Neopterygii,Teleostei, Neoteleostei, Acanthomorphata,Carangaria, Pleuronectiformes,Pleuronectoidei,Pleuronectidae Hippoglossoides.&&Animalia, Pisces
dbAMP_04362	GWKSVFRKAKKVGKTVGGLALDHYLG	26	Antibacterial, AntiGram_p, AntiGram_n, Antifungal	Q7SZH4	Eukaryota,Metazoa,Chordata,Craniata,Vertebrata, Euteleostomi, Actinopterygii,Neopterygii,Teleostei, Neoteleostei, Acanthomorphata,Carangaria, Pleuronectiformes,Pleuronectoidei,Pleuronectidae

					Hippoglossoides.&&Animalia, Pisces
dbAMP_04571	IFGAIAGFIKNIWX	14	Antimicrobial	Q6PP25	Viruses,ssRNA viruses,ssRNA negative-strand viruses, Orthomyxoviridae,Influenzavirus A.
dbAMP_04593	IGCGGGAVACQNYRQFCR	18	Antibacterial	P68577	Bacteria,Firmicutes,Bacilli,Bacillales,Bacillaceae, Bacillus.&&Bacteria
dbAMP_05155	KKWKKFIKKIGIGAVLTTPGAKK	23			Bacteria,Unclassified,Proteobacteria,Betaproteobacteria,Burkholderiales, Burkholderiaceae, Ralstonia, Ralstonia solanacearum
dbAMP_05285	KRKCPKTPFDNTPGAWFAHLILGC	24	AntiGram_p, Antimicrobial		Bacteria
dbAMP_10520	RKCNFLCKLKEKLRTVITSHIDKVLRPQG	29	AntiGram_p, AntiGram_n, Antifungal, Antimicrobial	M9MMP3	Eukaryota,Metazoa,Chordata,Craniata,Vertebrata, Euteleostomi, Amphibia,Batrachia, Anura, Neobatrachia, Ranoidea, Ranidae,Rana, Aquarana. &&Animalia, Amphibians
dbAMP_10834	RVKRFWPLVPVAINTVAAGINLYKAIRRK	29	Antibacterial, AntiGram_p, AntiGram_n	Q2IAL6	Eukaryota,Metazoa,Chordata,Craniata,Vertebrata, Euteleostomi, Archelosauria, Archosauria, Dinosauria, Saurischia,Theropoda,Coelurosauria, Aves, Neognathae, Galloanserae, Galliformes, Phasianidae, Phasianinae,Gallus.&&Animalia, Aves
dbAMP_10852	RWGKWFKKATHVGKHVGKAALTAYL	25	Antibacterial, AntiGram_p, AntiGram_n, Antifungal	Q7SZH1	Eukaryota,Metazoa,Chordata,Craniata,Vertebrata, Euteleostomi, Actinopterygii,Neopterygii, Teleostei,Neoteleostei, Acanthomorphata, Carangaria, Pleuronectiformes, Pleuronectoidei, Pleuronectidae, Limanda.&&Animalia, Pisces
dbAMP_11307	SWFSRTVHNVGNAVRKGIHAGQGVCSGLGL	30	Antibacterial, AntiGram_p, AntiGram_n		Eukaryota,Metazoa,Echinodermata, Echinoidea, Echinoida,Strongylocentrotidae,Strongylocentrotu, Strongylocentrotus droebachiensis &&Animalia, Echinoidea
dbAMP_11527	TNYGNGVGVPDAIMAGIIKLIFIFNIRQGYNFGKKAT	37	AntiGram_p		Bacteria,Unclassified,Proteobacteria,Deltaproteobacteria, Desulfuromonadales, Geobacteraceae, Geobacter, Geobacter sp. M18
dbAMP_11575	TTPACFTIGLGVGALFSAKFC	21	AntiGram_p, AntiGram_n, Antimicrobial, MammalianCells		Bacteria, Unclassified, Firmicutes, Bacilli, Lactobacillales, Enterococcaceae, Enterococcus, Enterococcus faecalis&&Bacteria
dbAMP_12362	SGSLSTFFRLFNRGGGWGHFFKKAAHVGKL	30	Antibacterial, AntiGram_p, Antibiofilm		Bacteria, Firmicutes, Bacilli, Lactobacillales, Streptococcaceae, Streptococcus,S. mutants

Supplementary Table 1C: All AMPs from dbAMP with additional sequence feature annotations from closest Swissprot match

dbAMP_ID	SEQUENCE	Length	Activity	Top_Blast_Match(Uniprot AC)	Top_Blast_Match (Uniprot ID)	E-value	Identity
dbAMP_00001	AAAAGSVWGAVNYTSDCNGECKRRGYKGGYCGSFANVNCWCET	43		P81544	DEFN_HELVI	2.00E-20	97
dbAMP_00004	AACARFIDDFCDTLTPNIYRPRDNGQRCYAVNGHRCDFTVFNTNNGGNPIRASTPNCKTVLRTAANRCPTGGRGKINPNAPFLFAIDPNDGDCSTNF	97	Antiviral, CancerCells Antitumour	P83811	CAP_COPCM	2.00E-65	100
dbAMP_00005	AACSDRAHGHICESFKSFCKDSGRNGVKLRANCKKTCGLC	40	Antibacterial, AntiGram_p, AntiGram_n	Q0MWV8	AURE_AURAU	1.00E-22	100
dbAMP_00006	AAEFPDFYDSEEQMGPHQEAEDEKDRADQRVLTEEEKKELENLAAMDLELQKIAEKFSQR	60	AntiGram_p, AntiGram_n, Antimicrobial	O35314	SCG1_RAT	9.00E-33	100
dbAMP_00024	AAFRGCWTKNYSPKPCL	17	Antibacterial, AntiGram_p	P83719	RACYT_RANTE	0.0007	75
dbAMP_00027	AAGGVKKPKKAAAAKKSPKKPKKPAAA	27	AntiGram_p	33	TOPO_DOM	-	-
dbAMP_00028	AAGMGFFGAR	10	AntiGram_p, AntiGram_n, Antifungal, Antimicrobial	-	-	-	-
dbAMP_00037	AAKIILNPKFRCKAAFC	17	AntiGram_n, EnzymeInhibitor	88	-	-	-
dbAMP_00043	AAKNKKEKGKKGASDCTEWTWGSCIPNSKDCGAGTREGTCKEETRKLKCKIPCNWKKAFGADCKYKFENWGECNATTGQKVRSGTLKKALYNADCQQTVEATKPCSLKTKSKSKGKKGKGKE	122	Antibacterial, AntiGram_p, AntiGram_n	P48530	MKA_XENLA	7.00E-82	100
dbAMP_00045	AAKPMGITCDLLSLWKVGHAACAAHCLVLGDVGGYCTKEGLCVCKE	46	Antibacterial AntiGram_n	O16136	DEF1_STOCA	5.00E-27	100
dbAMP_00046	AALKGCWTKSIPPKPCFGKR	20	Antibacterial, AntiGram_p, AntiGram_n, Antifungal EnzymeInhibitor	E7EKD9	ODRB1_ODOHA	3.00E-08	100
dbAMP_00047	AALKGCWTKSIPPKPCFRKR	20		E7EKD9	ODRB1_ODOHA	4.00E-07	95
dbAMP_00048	AALKGCWTKSIPPKPCSGKR	20	, AntiGram_p Antifungal Antimicrobial	E7EKD9	ODRB1_ODOHA	6.00E-07	95
dbAMP_00051	AALRGALRAVARVGKAILPHVAIANPYVRTPYVHNNP	37	Antibacterial, AntiGram_p, AntiGram_n, Antifungal	Q9PH97	AROQ_XYLFA	3.9	49
dbAMP_00052	AALRGCWTKSIPPKPCPGKR	20	Antibacterial, AntiGram_p, AntiGram_n, Antifungal	E7EKD9	ODRB1_ODOHA	2.00E-06	90
dbAMP_0005	AANFGPSVFTPEVHETWQKFLNVVVAALGK	33	AntiGram_n Antiparasitic	O13163	HBB_SILAS	5.00E-15	91

5	QYH						
dbAMP_0005 6	AAPCFCSGKPGRGDLWILRGTCPGGYGYTSN CYKWPNICCYPH	43	Antiviral	P0DMX6	BDS1_ANEVI	8.00E-27	100
dbAMP_0005 8	AAPRGGKGFFCKLFKDC	17	Antibacterial, AntiGram_p, AntiGram_n, Antifungal	-	-	-	-
dbAMP_0005 9	AAPTATVTPSSGLSDGTVVKVAGAGLQAGTA YDVGQCAWVDTGVLACNPADFSSVTADAN GSASTSLTVRRSFEGFLFDGTRWGTVDCTTA ACQVGLSDAAGNGPEGVAISFN	113	Antibacterial	P0A3R9	NCZS_STRCZ	1.00E-75	100
dbAMP_0006 7	AATAKKGAKKADAPAKPKKATKPKSPKKAAK KAGAKKGVKRAGKKGAKKTTKAKK	55	, AntiGram_p	35	-	-	
dbAMP_0007 1	AATKPKKAGAEAAPKKPAKKQTKKKPAKKAG GKKKPKRAGAKKAKK	46	, AntiGram_p	42	-	-	
dbAMP_0007 6	ACAAHCLLRGNRGGYCNGKG	20	Antibacterial, AntiGram_p	P10891	DEFI_PROTE	3.00E-07	100
dbAMP_0007 8	ACDFQQCWVTCQRQYSINFISARCNGDSCV CTFRT	35	Antifungal	55	-	-	-
dbAMP_0007 9	ACDTATCVTHRLAGLLSRSGGVVKNNFVPTN VGSKAF	37	Antibacterial AntiGram_n, Antifungal	P06881	CALCA_HUMAN	1.00E-20	100
dbAMP_0008 1	ACGILHDNCVYVPAQNPCCRGLQCRYGKCLV QV	33	Antiparasitic Antimalarial	P0C201	TXFK1_PSACA	2.00E-18	100
dbAMP_0008 3	ACHAHCQSVGRRGGYCGNFRMTCYCY	26	Antibacterial, AntiGram_p, AntiGram_n	B2D2C0	DEFI_ORNCO	4.00E-05	67
dbAMP_0008 4	ACIKNGGRCVASGGPPYCCSNYCLQIAGQSY GVCKKH	37	, AntiGram_p Antifungal	P81418	PAFP_PHYAM	1.00E-15	83
dbAMP_0008 6	ACLPNSCVSKGCCCGBSGYWCRQCGIKYTC	30	Antibacterial, AntiGram_p	P02885	SILU_RHIPU	2.00E-14	100
dbAMP_0008 7	ACNFQSCWATCQAQHSIYFRRAFCDRSQCK CVFVRG	36	Antibacterial, AntiGram_p Antifungal	P82321	TERN_PSEUS	1.00E-19	100
dbAMP_0008 8	ACQCPDAISGWTHTDYQCHGLENKMYRHV YAICMNGTQVYCRTEWGSSC	49	Antibacterial, AntiGram_p, AntiGram_n, Antifungal	P32004-3	L1CAM_HUMAN	0.46	46
dbAMP_0008 9	ACQFWSCNSSCISRGYRQGYCWGIQYKYCQ CQ	32	Antibacterial, AntiGram_p, AntiGram_n	Q6GU94	DEFL1_CENLI	3.00E-17	100
dbAMP_0009 0	ACSAG	5	Antibacterial, AntiGram_p, AntiGram_n Antiviral CancerCells	-	-	-	-
dbAMP_0009 1	ACVNQCPDAIDRFIVKDKGCHGVEKKYYKQV YVACMNGQHLYCRTEWGGPCQL	53	, AntiGram_p CancerCells	42	-	-	-
dbAMP_0010 3	ACYCRAPACIAGERRYGTCIYQGRLWAFCC	30	Antibacterial	P59665	DEF1_HUMAN	3.00E-15	97
dbAMP_0010	ACYCRIGACVSGERLTGACGLNGRIYRLCCR	31	Antibacterial, AntiGram_p, AntiGram_n,	Q62714	DEF4_RAT	2.00E-15	100

4			Antifungal, Antiviral				
dbAMP_0010 5	ACYCRIPACFAGERRYGTCFYLGRVWAFCC	30	AntiGram_p, AntiGram_n, Antifungal Antimicrobial AntiMRSA	P60030	DEF1_MACMU	8.00E-16	97
dbAMP_0010 6	ACYCRIPACIAGEAAYGTCIYQGALWAFCC	30	Antibacterial	P59665	DEF1_HUMAN	3.00E-13	90
dbAMP_0010 7	ACYCRIPACIAGERRAGTCAYQGRAWAACC	30	Antimicrobial	P59665	DEF1_HUMAN	8.00E-12	87
dbAMP_0010 8	ACYCRIPACIAGERRAGTCIYQGRLWAACC	30	Antimicrobial	P59665	DEF1_HUMAN	2.00E-13	93
dbAMP_0010 9	ACYCRIPACIAGERRAGTCIYQGRLWAFCC	30	Antibacterial	P59665	DEF1_HUMAN	8.00E-15	97
dbAMP_0011 0	ACYCRIPACIAGERRYATCIYQGRLWAFCC	30	Antibacterial	P59665	DEF1_HUMAN	3.00E-15	97
dbAMP_0011 2	ACYCRIPACIAGERRYGTCAYQGRAWAFCC	30	Antimicrobial	P59665	DEF1_HUMAN	2.00E-14	93
dbAMP_0011 3	ACYCRIPACIAGERRYGTCAYQGRLWAFCC	30	Antimicrobial	P59665	DEF1_HUMAN	3.00E-15	97
dbAMP_0011 4	ACYCRIPACIAGERRYGTCIAQGRLWAFCC	30	Antibacterial	P59665	DEF1_HUMAN	8.00E-15	97
dbAMP_0011 5	ACYCRIPACIAGERRYGTCIYAGRLWAFCC	30	Antibacterial	P59665	DEF1_HUMAN	4.00E-15	97
dbAMP_0011 6	ACYCRIPACIAGERRYGTCIYQGALWAFCC	30	Antibacterial	P59665	DEF1_HUMAN	4.00E-15	97
dbAMP_0011 7	ACYCRIPACIAGERRYGTCIYQGRLAAFCC	30	Antibacterial	P59665	DEF1_HUMAN	2.00E-14	97
dbAMP_0011 8	ACYCRIPACIAGERRYGTCIYQGRLWAACC	30	Antibacterial	P59665	DEF1_HUMAN	1.00E-14	97
dbAMP_0011 9	ACYCRIPACIAGERRYGTCIYQGRLWAFCC	30	Antibacterial, AntiGram_p, AntiGram_n, Antifungal, Antiviral, Antiparasitic, AntiHIV,WoundHealing,Chemotactic,EnzymeInhibitor,CancerCells	P59665	DEF1_HUMAN	4.00E-16	100
dbAMP_0012 0	ACYCRIPACIAGERRYGTCIYQGRLWAXCC	30	Antimicrobial	DEF1_HUMAN	8.00E-15	97	-
dbAMP_0012 1	ACYCRIPACLAGERRYGTCFYLGRVWAFCC	30	Antibacterial, AntiGram_p Antifungal	P60030	DEF1_MACMU	3.00E-16	100
dbAMP_0012 2	ACYCRIPACLAGERRYGTCFYLRRVWAFCC	30	Antibacterial Antifungal	P60032	DEF8_MACMU	5.00E-16	100
dbAMP_0012 3	ACYCRIPACLAGERRYGTCFYMGRVWAFCC	30	Antibacterial, AntiGram_p, AntiGram_n, Antifungal	P60030	DEF1_MACMU	5.00E-16	97
dbAMP_0012	ACYCRIPACLAGERRYGTCFYRRRVWAFCC	30	Antibacterial, AntiGram_p Antifungal	P60031	DEF3_MACMU	5.00E-16	100

4							
dbAMP_0012 7	ADDGNPLEECFRETDYEEFLEIAKNGLSATSN PKHVVIVGAGMSGLSAAYVLANAGHQVTVL EASKRAGGRVRTYRNDKEGWYANLGPMRL PEKHRIVREYIRKFGLQLNEFSQENENAWYFI KNIRKRVGEVNKDPGVLEYPVKPSEVGKSAG QLYEESLQKAVEELRRTNCSYMLNKYDTYSTK EYLLKEGNLSPGAVDMIGDLLNEDSGYYVSFI ESLKHDDIFAYEKRFDEIVGGMDKLPTSMYQ AIQEKV	255	Antibacterial, AntiGram_p, AntiGram_n Antiparasitic CancerCells	B5AR80	OXLA_BOTPA	0	100
dbAMP_0012 8	ADDGNPLEECFRETDYEEFLEIAKNGLSATSN PKHVVIVGAGMSGLSAAYVLANAGHQVTVL EASKRAGGRVRTYRNDKEGWYANLGPMRL PEKHRIVREYIRKFGLQLNEFSQENENAWYFI KNIRKRVGEVNKDPGVLEYPVKPSEVGKSAG QLYEESLQKAVEELRRTNCSYMLNKYDTYSTK EYLLKEGNLSPGAVDMIGDLLNEDSGYYVSFI ESLKHDDIFAYEKRFDEIVGGMDKLPTSMYQ AIQEKVRLNVRVIKIQQDVKEVTVTYQTSAKE TLSVTADYVIVCTTSRAARRIKFEPPLPPKKAH ALRSVHYRSGTKIFLTCTKKFWEDDGIHGGKS TTDLPSRFIYYPNHNFPSGVGVIIAYGIGDDA NFFQALDFKDCGDIVINDLSLIHQLPKEEIQAF CRPSMIQRWSLDKYAMGGITTFTPYQFQHF SEALTAPVDRIYFAGEYTAQAHGWIDSTIKSG LTAARDVNRASE	485		100	-	-	-
dbAMP_0012 9	ADDKNPLEEAFREADYEVFLEIAKNGL	27	Antibacterial	P0C2D3	OXLA_ERIMA	4.00E-12	100
dbAMP_0013 1	ADDKNPLEECFREDDYEEFLEIAKNGLEGWY ANLGPMRYPVKPSEEGKHDDIFAYEKFDEIV GGMDKKFWEDDGIHGGKETFCYSPMIQKPY QFQHFSEALTAPVGR	107	Antibacterial, AntiGram_p, AntiGram_n	P81375	OXLA_MACLB	2.00E-76	100
dbAMP_0013 2	ADDKNPLEEFRETNYEVFLEIAKNGLKATSNP KRVVIVGAGMAGLSAAY	49	Antibacterial AntiGram_n CancerCells Antitumour	P0C2D1	OXLA_BOTPI	1.00E-29	100
dbAMP_0013 3	ADDRCERMCQRYHDRREKKQCMKGCRYG	28	Antifungal	B3EWR6	AMP_ECHCG	7.5	46
dbAMP_0013 5	ADDRNPLAECFQENDYEEFLEIARNGLKATS NPKHVVIVGAGMAGLSAAYVLAGAGHQVT VLEASERPGGRVRTYRNEEAGWYANLGPMR LPEKHRIVREYIRKFDLRLNEFSQENDNAWYF	498	Antibacterial	P81382	OXLA_CALRH	0	100

	IKNIRKKVGEVKKDPGLLKYPVKPSEAGKSAG QLYEESLGKVVEELKRTNCSYILNKYDTYSTKE YLIKEGDLSPGAVDMIGDLLNEDSGYYVSFIES LKHDDIFAYEKRFDEIVDGMDKLPTAMYRDI QDKVHFNAQVIKIQQNDQKVTVVYETLSKET PSVTADYVIVCTTSRAVRLIKFNPPLLPKKAHA LRSVHYRSGTKIFLTCTTKFWEDDGIHGGKST TDLPSRFIYYPNHNFTNGVGVIIAYGIGDDAN FFQALDFKDCADIVFNDLSLIHQLPKKDIQSFC YPSVIQKWSLDKYAMGGITTFTPYQFQHFSD PLTASQGRIYFAGEYTAQAHGWIDSTIKSGLR AARDVNLASENPSGIHLSNDNEL						
dbAMP_0013 7	ADDRNPLEECFRETDYEEFLEIAKNGLSTTSNP KRVVIVGAGMSGLSAAYVLANAGHQVTVLE ASERAGGRVKTYRNEKEGWYANLGPMRLPE KHRIVREYIRKFDLQLNEFSQENENAWYFIKN IRKRVGEVNKDPGVLEYPVKPSEVGKSAGQL YEESLQKAVEELRRTNCSYMLNKYDTYSTKEY LLKEGNLSPGAVDMIGDLLNEDSGYYVSFIES LKHDDIFAYEKRFDEIVGGMDKLPTSMYQAI QEKV	255	Antibacterial	Q6TGQ8	OXLA_BOTMO	0	100
dbAMP_0013 8	ADDRNPLEECFRETDYEEFLEIARNGLKATSN PKHVVIVGAGMSGLSAAYVLSGAGHQVTVL EASERAGGRVRTYRNDKEGWYANLGPMRLP EKHRIVREYIRKFGLQLNEFSQENDNAWYFIK NIRKRVGEVKKDPGVLKYPVKPSEEGKSAGQ LYEESLGKVVEELKRTNCSYILNKYDTYSTKEYL LKEGNLSPGAVDMIGDLMNEDSGYYVSFPES LRHDDIFAYEKRFDEIVGGMDKLPTSMYRAIE EKV	255	Antibacterial	Q90W54	OXLA_GLOBL	0	100
dbAMP_0013 9	ADDRNPLEQCFRETDYEEFLEIARNNLKATSN PKHVVIVGAGMAGLSAAYVLSGGGHQVTV	61	Antibacterial, AntiGram_p, AntiGram_n Antiparasitic	P0C2D2	OXLA_CRODC	2.00E-40	100
dbAMP_0014 2	ADDRRRPLEECFREADYEEFLEIAKNGLQRTS NPKRVVVVGAGMAGLSAAYVLAGAGHQVT LLEASERVGGRVNTYRNEKDGWYVNLGPMR LPERHRIIREYIRKFGLELNEFIQENDNAWYFI KNIRKRVSEVKKDPGVFKYPVKPSEEGKSASQ LYRESLQKVIEELKRTNCSYILNKYDTYSTKEYLI KEGNLSPGAVDMIGDLLNEDSSYYLSFIESLKS DDIFSYEKRFDEIVGGFDQLPRSMYQAIAEK	255	Antibacterial	Q4JHE1	OXLA_PSEAU	0	100

dbAMP_0014 3	ADNFSLHDALSGSGNPNPQGWPGAWGNQ PAGAGGYPGASYPGAYPGQAPPGAYPGQA PPGAYPGAPGAYPGAPAPGVYPGPPSGPGA YPSSGQPSATGAYPATGPYGAPAGPLIVPYNL PLPGGVVPRMLITILGTVKPNANRIALDFQRG NDVAFHFNPRFNENNRRVIVCNTKLDNNW GREERQSVFPFESGKPFKIQVLVEPDHFKVAV NDAHLLQYNHRVKKLNEISKLGISGDIDLTSAS YTMI	249	Antibacterial Antifungal	P17931	LEG3_HUMAN	2.00E-179	100
dbAMP_0014 4	ADNKNPLEECFRETNYEEFLEIAR	24	Antibacterial, AntiGram_p, AntiGram_n	P0C2D6	OXLA_PROMU	3.00E-10	100
dbAMP_0014 5	ADPTFGFTPLGLSEKANLQIMKAYD	25	Antifungal	P86520	SBTXA_SOYBN	9.00E-12	100
dbAMP_0014 7	ADRDQYELL	9		-	-	-	-
dbAMP_0014 8	ADRGWIKTLTKDCPNVISSICAGTIITACKNCA	33	Antibacterial, AntiGram_p, AntiGram_n	H2A7G5	LANA_STRMD	2.00E-17	100
dbAMP_0015 1	ADTLACRQSHQSCSFVACRAPSVDIGTCRGG KLKCCKWAPSS	42	Antibacterial, AntiGram_p, AntiGram_n	Q6QLR1	GLL9_CHICK	1.00E-23	98
dbAMP_0016 0	AEETSFVFSKFKPLEPNLILQGDALVTVAGVL QLTNVDKNGVPEPSSLGRATYSAPINIWDSA TGLVASFATSFRFTIYAPNIATIADGLAFFLAPV ASAPDSGGGFLGLFDSAVSGSTYQTVAVEFD TYENTVFTDPPYTHIGFDVNSISSIKTVKWSLA NGEAAKVLITYNSAVKLLVASLVYPSSKTSFILA DIVDLSSVLPEWVRVGFSAATGASGGKIETH DVFSWSFASKLAGXXTKDSSFLDGG	251	Antifungal	P86352	LECA_SPAPA	2.00E-177	100
dbAMP_0016 1	AEETSFVFSKFKPLEPNLILQGDALVTVAGVL QLTNVDSNGVPEPSSLGRATYSAPINIWDSA TGLVASFATSFRFTIYAPNIATIADGLAFFLAPV ASAPDSGGGFLGLFDSAVGDTTYQTVAVEFD TYENTVFTDPPYTHIGFDVNSISSIKTVKWSLA NGEAAKVLITYNSAVKLLVASLVYPSSKTSFILA DIVDLSSVLPEWVRVGFSAATGASKGYIETHD VFSWSFASKLAG	239	Antifungal	P86353	LECB_SPAPA	1.00E-170	100
dbAMP_0016 6	AELRCLCIKTTSGIHPKNIQSLEVIGKGTHCNQ VEVIATLKDGRKICLDPDAPRIKKIVQKKLAGD ESAD	70	Antibacterial	P02775	CXCL7_HUMAN	3.00E-45	99
dbAMP_0016 7	AELRCMCIKTTSGIHPKNIQSLEVIGKGTHCN QVEVIATLKDGRKICLDPDAPRIKKIVQKKLAG	66	Antibacterial, AntiGram_p, AntiGram_n, Antifungal	P02775	CXCL7_HUMAN	7.00E-43	100

	D						
dbAMP_00168	AELRCMCIKTTSGIHPKNIQSLEVIGKGTHCNQVEVIATLKDGRKICLDPDAPRIKKIVQKKLAGDES	68	Antibacterial, AntiGram_p, AntiGram_n, Antifungal	P02775	CXCL7_HUMAN	2.00E-44	100
dbAMP_00171	AERVGAGAPVYL	12	Antibacterial, AntiGram_p Antifungal	Q9BTM1	H2AJ_HUMAN	0.65	100
dbAMP_00172	AEVAPAPAAAAPAKAPKKKAAAKPKKAGPS	30	Antibacterial, AntiGram_p, AntiGram_n	P84408	H1_SALSA	6.00E-09	100
dbAMP_00174	AFFARLLASVRAAVKAFAKKPRLIGLSTLL	30	Antibacterial, AntiGram_p, AntiGram_n, Antifungal	58	-	-	-
dbAMP_00184	AFGCPFDQGTCHSHCRSIRRRGRRCASFAKRTCTCYQK	38	Antifungal	68	-	-	-
dbAMP_00185	AFKLLGRIIHHVGNFVYGFSHVF	23	Antibacterial, AntiGram_p, AntiGram_n, Antifungal	P80713	CLAVD_STYCL	2.00E-09	100
dbAMP_00187	AFPPPNVPGPRFPPPNFPGPRFPPPNFPGPRFPPPNFPGPRFPPPNFPGPPFPPPIFPGPWFPPPPPFRPPPFGPPRFP	79	Antibacterial, AntiGram_p, AntiGram_n	P51524	PF11_PIG	2.00E-35	100
dbAMP_00191	AFTCHCRRSCYSTEYSYGTCTVMGINHRFCCL	32	Antibacterial Antifungal, Antiviral	Q01524	DEF6_HUMAN	3.00E-18	100
dbAMP_00192	AFTCHCRRSCYSTEYSYGTCTVMGINWRFCCL	32	Antibacterial	DEF6_HUMAN	4.00E-17	97	-
dbAMP_00195	AGANDLCQECEDIVHLLTKMTKEDAFQDTIRKFLEQECDILPLKLLVPRCRQVLDVYLPLVIDYFQGQIKPKAICSHVGLC	81	Antibacterial, AntiGram_p, AntiGram_n	P22355	PSPB_RAT	2.00E-51	100
dbAMP_00196	AGCIKNGGRCNASAGPPYCCSSYCFQIAGQSYGVCKNR	38	Antibacterial, AntiGram_p Antifungal	P81418	PAFP_PHYAM	9.00E-22	100
dbAMP_00197	AGDDETLLKPVLNSLDNLVSGL	22		P86511	ROT31_LITRO	8.00E-08	100
dbAMP_00200	AGECVQGRCPSGMCCSQFGYCGRGPKYCGR	30	Antifungal	Q5I2B2	AMP_AMARE	4.00E-16	100
dbAMP_00203	AGETCVGGTCNTPGATCSWPVCTRNGLPV	29	Antibacterial	P56254	KAB1_OLDAF	1.00E-12	96
dbAMP_00204	AGETHTVMINHAGRGAPKLVVGGKKLS	27	Antifungal	P84995	GANOD_GANLU	4.00E-12	100
dbAMP_00205	AGFAAQAAASLAPVAAQQL	19	Antibacterial, AntiGram_p Antifungal	-	-	-	-
dbAMP_00206	AGFAAQAAASLAPVAIQQL	19	Antibacterial, AntiGram_p Antifungal	-	-	-	-
dbAMP_00208	AGFRKRFNKLVKKVKHTIKETANVSKDVAIVAGSGVAVGAAMG	43	, AntiGram_p	77	-	-	-

dbAMP_00209	AGFSMDKANSEACRDGLRAVMECRNVTHLLQQELTEAQKGFQDVEAQAATCNHTVMALMASLDAEKAQGQKKVEELEGEITTLNHKLQDASAEVERLRRENQVLSVRIADKKYYPSSQDSS	121	Antiviral	Q10589-2	BST2_HUMAN	1.00E-80	100
dbAMP_00210	AGFVLKGYTKTSQ	13	Antibacterial AntiGram_n	P82239	SAL2B_ONCMY	0.075	100
dbAMP_00214	AGLQFPVGRIGRLLRK	16	AntiGram_p, AntiGram_n, Antifungal	94	-	-	-
dbAMP_00215	AGRGKQGGKVRAKAKTRSSRAGLQFPVGRVHRLLRKGNY	39	Antibacterial, AntiGram_p, AntiGram_n, Antifungal	P55897	H2A_BUFGR	4.00E-20	100
dbAMP_00217	AGWGSIFKHIFKAGKFIHGAIQAHND	26	Antibacterial, AntiGram_p, AntiGram_n, Antifungal	-	-	-	-
dbAMP_00219	AGYLLGKINLKALAALAKKIL	21	Antibacterial AntiGram_n	86	-	-	-

Supplementary Table 1D: summary and raw output for Abeta42 PSIBLAST searches against dbAMP

ID1_Query	ID_Target	E-value	Score	Range Query	Range Target	Coverage
sp|P05067|672-713	dbAMP_07565	9.6	54	01:13	103:115	0.2857

sp|P05067|672-713	dbAMP_08227	4.7	58	27:38:00	40:51:00	0.2619
sp|P05067|672-713	dbAMP_08871	7.3	44	25:42:00	54:71	0.4048
sp|P05067|672-713	dbAMP_00283	3.7	58	27:38:00	22:33	0.2619
sp|P05067|672-713	dbAMP_08199	0.44	29	08:41	05:38	0.7857
sp|P05067|672-713	dbAMP_00972	6.00E-26	100	01:40	01:40	0.9286
sp|P05067|672-713	dbAMP_00973	4.00E-27	100	01:42	01:42	0.9762
sp|P05067|672-713	dbAMP_10113	3.7	40	27:41:00	03:17	0.3333
sp|P05067|672-713	dbAMP_01287	10	53	25:39:00	47:61	0.3333
sp|P05067|672-713	dbAMP_06900	3.2	38	22:42	14:33	0.4762
